# Factors associated with true‐positive and false‐positive diagnoses of behavioural variant frontotemporal dementia in 100 consecutive referrals from specialist physicians

**DOI:** 10.1111/ene.70036

**Published:** 2025-01-14

**Authors:** Joshua Flavell, Emily G. M. Ahern, Benignus Logan, Thomas B. Shaw, Robert J. Adam, Caitlin A. T. McElligott, Peter J. Nestor

**Affiliations:** ^1^ The Queensland Brain Institute The University of Queensland Brisbane Queensland Australia; ^2^ The Mater Memory and Cognitive Disorders Clinic The Mater Hospital Brisbane Queensland Australia; ^3^ Centre for Health Services Research The University of Queensland Brisbane Queensland Australia; ^4^ Royal Brisbane and Women's Hospital and Surgical Treatment and Rehabilitation Service (STARS), Metro North Hospital and Health Service Brisbane Queensland Australia; ^5^ Centre for Advanced Imaging The University of Queensland Brisbane Queensland Australia; ^6^ School of Electrical Engineering and Computer Science The University of Queensland Brisbane Queensland Australia; ^7^ Centre for Clinical Research The University of Queensland Brisbane Australia

**Keywords:** frontotemporal dementia, misdiagnosis, neuroimaging, neuropsychology

## Abstract

**Background:**

The behavioural variant of frontotemporal dementia (bvFTD) is a challenging diagnosis due to overlapping symptoms with psychiatric and other neurological conditions. Accordingly, misdiagnosis is common. The present study aimed to identify clinical factors contributing to misdiagnoses of bvFTD by specialist physicians.

**Methods:**

We retrospectively analysed 100 consecutive referrals by specialist physicians (primarily psychiatrists, neurologists and geriatricians) to a tertiary cognitive disorders clinic specializing in frontotemporal lobar degenerative disorders. Patients were included if the referring specialist suspected bvFTD or if bvFTD was confirmed as the final diagnosis. Diagnostic factors were assessed by comparing the initial referral information with final clinical diagnoses.

**Results:**

Of the 100 patients, 34 were true‐positive and 66 were false‐positive for bvFTD. False‐positive diagnoses were often based on misinterpretation of neuroimaging, particularly nuclear imaging (FDG‐PET and HMPAO‐SPECT), where subjective interpretation errors led to incorrect bvFTD diagnoses in 32 patients. Cognitive testing also contributed to misdiagnosis, with formal neuropsychological testing incorrectly leading to a bvFTD diagnosis in 20 patients. Patients with prior psychiatric histories were more likely to be misdiagnosed. Observable behavioural features of bvFTD and physical neurological signs were significantly more prevalent in true‐positive patients.

**Conclusions:**

Misinterpretation of neuroimaging and cognitive testing, in particular formal neuropsychological testing, significantly contributed to false‐positive bvFTD diagnoses. Physicians should be cautious not to over‐interpret neuroimaging and neuropsychology studies and be wary of patients with prior psychiatric histories. In contrast, greater weight should be placed on objective clinical observations of behavioural signs of bvFTD and the emergence of physical neurological signs.

## INTRODUCTION

The behavioural variant of frontotemporal dementia (bvFTD) is a common form of young‐onset dementia, but the diagnosis can be unstable. It is often initially misdiagnosed as a psychiatric disorder [1] while some diagnosed with bvFTD will subsequently have their diagnosis changed [2]. The Frontotemporal Dementia Consortium criteria, also referred to as the ‘Rascovsky criteria’, categorizes the clinical features of bvFTD by integrating behavioural symptoms, neuropsychological, neuroimaging, histopathological and genetic information [3]. Nonetheless, detecting the behavioural features of bvFTD and discriminating them from other disorders can be challenging.

Patients characteristically lack insight into their behavioural changes, making them unreliable historians [4, 5]. This means that identification of the clinical phenotype of bvFTD heavily depends on an informant—whose symptom reporting may be subjective. For instance, because informants usually lack prior experience with bvFTD, they may struggle to recognize when behaviours, such as disinhibition, lack of empathy or preference for sweet foods, reach a level indicative of a neurodegenerative disease. These factors likely contributed to the finding in one study that physicians had an accuracy of only 27% in correctly identifying bvFTD [6]. The study examined the referral symptomatology and demographic details associated with true‐positive and false‐positive diagnoses but did not elaborate on how other clinical factors impacted the diagnosis.

The present study aimed to identify factors leading to true‐positive and, particularly, false‐positive diagnoses of bvFTD by examining the referrals from specialist physicians to a tertiary cognitive disorders clinic. Specifically, we sought to examine the types of evidence that had been used by physicians in reaching their diagnosis with a view to identifying whether or not such evidence ultimately proved reliable. Such analyses might offer potential to improve diagnostic accuracy by highlighting pitfalls in the diagnostic process.

## METHODS

### Study design

The medical records of patients referred to the Mater Hospital Memory and Cognitive Disorders Clinic were reviewed starting from the clinic's inception with the aim of identifying *n* = 100 consecutively referred patients in whom either the final diagnosis was bvFTD or where this diagnosis had been suggested by the referring specialist physician regardless of the final diagnosis. The clinic, founded in 2018, is tertiary referral only and specialized in the assessment and management young‐onset dementias with a particular focus on frontotemporal lobar degeneration (FTLD). The specialist physicians who refer to the clinic are typically neurologists, psychiatrists or geriatricians.

### Referral information from specialists

The clinical information sent from referring specialists along with the results of any investigations that they had undertaken prior to referral were scrutinized to understand the rationale for suspecting bvFTD in each patient. As one would expect, there was considerable variability in the types of investigations and assessments done by referring physicians meaning that the numbers for each type of investigation (e.g. neuropsychology and neuroimaging) varied.

### The mater clinic assessment

At the Mater clinic, patients routinely underwent a detailed clinical assessment and separate informant interview. This included the Addenbrooke's Cognitive Examination III (ACE‐III) [7]; an in‐house informant‐behavioural questionnaire (IBQ); and the Hospital Anxiety and Depression Scale [8] or the 30‐item Geriatric Depression Scale [9].

The IBQ assesses the symptoms associated with all of the major syndromes seen in the degenerative dementias. Each item on the questionnaire is rated as present either (0) never, (1) rarely, (2) sometimes, (3) often or (4) constantly. A score of 2 or more was used as the threshold to define the presence of a behavioural symptom. The IBQ includes questions probing all of the frontotemporal behavioural features of the Rascovsky criteria, namely disinhibition, apathy, lack of empathy, stereotypical and perseverative behaviours, and, hyperorality and eating behaviours. It also assesses symptoms related to impairments in cognitive domains; functional abilities, including questions about basic and instrumental activities of daily living; and psychiatric symptoms, including questions probing for delusions, hallucinations, depression and anxiety. The IBQ is available upon request from the authors. IBQ data were available in *n* = 94/100 patients, with *n* = 6 patients missing data primarily due to a lack of an informant attending the clinic in person.

Results from the ACE‐III were extracted to define the cognitive profile of bvFTD—executive dysfunction with sparing of memory and visuospatial abilities. Executive function was assessed by phonemic fluency; memory by delayed name and address recall; and visuospatial abilities by the ACE‐III visuospatial sub‐score. Cut‐off scores to define impairment were defined by 1.5 standard deviations from the mean scores of *n* = 35 age‐matched healthy controls, as reported in a previous study [10]. ACE‐III data were available in *n* = 97/100 patients, with *n* = 2 true‐positive bvFTD patients being untestable because of advanced disease and *n* = 1 false‐positive patient missing data due to test refusal.

Additionally, the clinical notes of the initial assessment were reviewed for observable behaviours and physical neurological signs of FTLD. Observable behaviour features of bvFTD included instances where the examining physician could directly observe characteristic bvFTD behaviours without having to rely on informant testimony (e.g. verbal stereotypies, social inappropriateness and utilization behaviour). Physical neurological signs of FTLD included motor features of progressive supranuclear palsy (PSP), motor neuron disease (MND) and corticobasal syndrome (CBS).

Additional investigations and longitudinal clinical follow‐up varied according to clinical need. These assessments could include, where indicated: nuclear imaging with ^18^F‐fluorodeoxyglucose positron emission tomography (FDG‐PET); structural magnetic resonance imaging (MRI); neuropsychological testing; genetic testing where suggestive of a familial disorder or unclear; and cerebrospinal fluid examinations particularly where inflammatory diseases or Alzheimer's disease were considered possible.

### Diagnostic classification

BvFTD was diagnosed using the Rascovsky criteria, while other FTLD and neurological disorders were diagnosed according to established criteria, such as the National Institute on Aging and Alzheimer's Association for Alzheimer's disease [11]. Primary psychiatric disorders were diagnosed by a psychiatrist using the Diagnostic and Statistical Manual of Mental Disorders, Fifth Edition (DSM‐5) [12].

Patients were classified as possible, probable or definite bvFTD, or, had the diagnosis excluded, based on the Rascovsky criteria. Those with possible bvFTD at initial assessment and no clear alternative diagnosis were followed up longitudinally to either confirm or rule out bvFTD (maximum follow‐up to reach diagnosis was 63 months).

In total, *n* = 102 consecutive patients were identified for the study comprising *n* = 100 in whom the referring physician had suggested bvFTD and *n* = 2 in whom the final diagnosis was probable bvFTD but the referrer had not specified any diagnosis. Of the *n* = 100 with putative bvFTD proposed by the referrer, *n* = 2 were excluded because insufficient follow‐up at the time of analysis meant they remained diagnostically indeterminate, classified only as possible bvFTD.

The ‘true‐positive’ cohort included: (A) definite bvFTD—the clinical phenotype of bvFTD and either histopathological confirmation or a known pathogenic gene mutation; (B) probable bvFTD—the clinical phenotype of bvFTD and evidence of progressive focal frontal/temporal degeneration on serial neuroimaging; (C) ‘bvFTD+’—patients who initially presented with the clinical phenotype of bvFTD but later developed another FTLD‐related syndrome (e.g. MND and PSP).

### Data collection

Patient demographics, including sex, age, education level, symptom duration, psychiatric and neurological history, and psychiatric medication use at referral, were analysed to assess differences between true‐positive and false‐positive patients. Demographic data were available for all patients, except for education level, which was available for *n* = 93/100.

The rationale for suspecting bvFTD in the referral letters was categorized into structural neuroimaging, nuclear imaging, cognitive testing, family history and diagnostic dilemmas (i.e. ambiguity in clinical features). This included examining investigations performed by referring physicians, such as reports of neuroimaging and neuropsychological assessments. The neuropsychology assessments were reviewed for evidence of executive dysfunction; sparing of memory and visuospatial abilities; and, the combination of both (this being the purported cognitive profile of bvFTD in the Rascovsky criteria). No neuropsychology reports assessed social cognition, and thus, this was not included in the results.

### Data analysis

The data analysis contrasted true‐positive and false‐positive cohorts. Chi‐squared (*χ* ^2^) test was used for nominal data; *t*‐test for normally distributed numerical data; and Mann–Whitney *U*‐test for non‐normally distributed numerical data. Nominal data were presented in proportions (e.g. percentage), normally distributed numerical data were presented as mean ± standard deviation, and non‐normally distributed numerical data were reported as median (range).

## RESULTS

### Diagnostic outcomes

**FIGURE 1:**
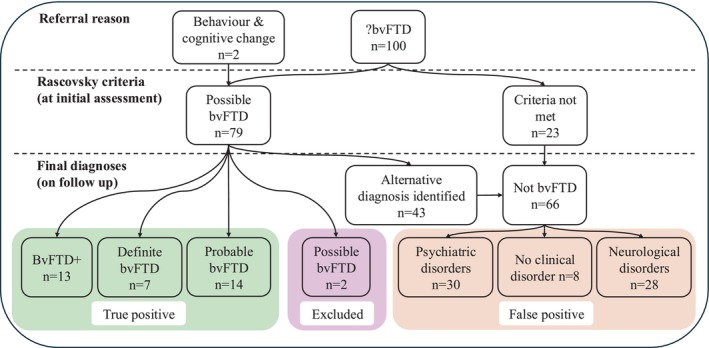
Flow chart of diagnostic outcomes. bvFTD, behavioural variant frontotemporal dementia; BvFTD+, patients who met Rascovsky criteria for possible bvFTD but also another clinical presentation of frontotemporal lobar degeneration. Two patients referred without a suspected diagnosis were true‐positive bvFTD cases on follow‐up.

Among the final 100 patients included in the study, *n* = 34 were true‐positive patients and *n* = 66 false‐positive patients. (Figure 1). The true‐positive cohort included *n* = 7 definite bvFTD, *n* = 14 probable bvFTD and *n* = 13 bvFTD+. The false‐positive cohort included *n* = 30 psychiatric disorders, *n* = 28 neurological disorders and *n* = 8 who did not meet criteria for any clinical disorder. This last group had either psychosocial problems (e.g. employment or relationship issues) or sub‐clinical psychiatric symptoms (e.g. sub‐clinical features of autism) that explained the presenting behavioural symptoms. Often these patients, or their informant, minimized these psychosocial issues or they only became apparent on follow‐up (Table 1).

**TABLE 1:** Diagnostic outcomes.

Diagnostic category	Total (*n*)	Final diagnoses
True‐positive patients (*n* = 34)		
Definite bvFTD	7	C9orf72 mutations (*n* = 5) Histopathology confirmed (*n* = 2)
Probable bvFTD	14	With progressive atrophy (*n* = 14)
BvFTD+	13	+ Progressive supranuclear palsy (*n* = 5) + Primary progressive aphasia (*n* = 4) + Corticobasal syndrome (*n* = 3) + Motor neuron disease (*n* = 1)
Primary psychiatric disorders (*n* = 30)		
Mood disorders	12	Major depressive disorder (*n* = 6) Bipolar disorder type 1 (*n* = 5) Persistent depressive disorder (*n* = 1)
Neurodevelopmental disorders	5	Autism spectrum disorder (*n* = 3) Intellectual disability (*n* = 2)
Psychotic disorders	4	Schizophrenia (*n* = 2) Schizoaffective disorder (*n* = 1) Drug‐induced psychosis (*n* = 1)
Anxiety disorders	2	Generalized anxiety disorder (*n* = 1) Adjustment disorder with anxiety (*n* = 1)
Other disorders	7	Post‐traumatic stress disorder (*n* = 3) Obsessive compulsive disorder (*n* = 2) Alcohol use disorder (*n* = 1) Functional neurological disorder (*n* = 1)
Neurological disorders (*n* = 28)		
Degenerative dementias	19	Typical Alzheimer's disease (*n* = 14) Posterior cortical atrophy (*n* = 1) Dementia with Lewy bodies (*n* = 1) Suspected LATE* (*n* = 1) Huntington's disease dementia (*n* = 1) CSF‐1R mutation leukoencephalopathy (*n* = 1)
Vascular disorders	2	Stroke (*n* = 1) Cerebral vasculitis (*n* = 1)
Other disorders	7	Frontal lobe tumours (*n* = 2) Chronic migraine (*n* = 2) Autoimmune encephalitis (*n* = 1) Congenital abnormality (*n* = 1) Traumatic brain injury (*n* = 1)
No clinical disorder (*n* = 8)		
Psychosocial issues	5	Relationship breakdown (*n* = 2) Forensic issues (*n* = 2) Employment issues (*n* = 1)
Sub‐clinical psychiatric symptoms	3	Sub‐clinical autism spectrum disorder (*n* = 2) Sub‐clinical depression (*n* = 1)

* Limbic‐predominant age‐related TDP‐43 encephalopathy.

### Demographic details

False‐positive bvFTD patients were more likely to have a past history of a psychiatric disorder (*χ* ^2^ = 7.289, *p* < 0.05) and to have used psychiatric medications (*χ* ^2^ = 14.842, *p* < 0.01) compared with true‐positive bvFTD patients. There were no significant differences between groups in terms of sex, age, education level, symptom duration or history of a neurological disorder (Table 2).

**TABLE 2:** Comparison of demographic details between true‐ and false‐positive patients.

Demographic	True positives	False positives	Test	*P*‐value
Age at symptom onset (years)	59.4 ± 11.8	57.7 ± 10.5	*t* = 0.75	0.46
Age at clinic presentation (years)	64.4 ± 10.8	63.0 ± 9.1	*t* = 0.69	0.49
Education level* (years)	12.0 (9.0–24.0)	12.0 (9.0–26.0)	*U* = 856.50	0.54
Symptom duration (years)	3.0 (1.0–22.0)	3.0 (0.8–55.0)	*U* = 1098.50	0.86
Sex (M:F)	20:14	43:23	*χ* ^2^ = 0.39	0.54
Neurological history (%)	41.2	50.0	*χ* ^2^ = 0.70	0.40
Psychiatric history (%)	47.1	74.2	*χ* ^2^ = 7.29	0.01
Psychiatric medication at referral (%)	38.2	77.3	*χ* ^2^ = 14.84	<0.01

* Data available for *n* = 93 patients.

Abbreviations: F, female; M, male.

#### Reasons for a false‐positive diagnosis

Among the false‐positive bvFTD patients, *n* = 45 had a singular, clear justification for suspecting bvFTD, while the remaining *n* = 21 had multiple reasons contributing to the incorrect diagnosis. The commonest reasons for a specialist physician to incorrectly suspect bvFTD was nuclear imaging, followed by cognitive testing—including formal neuropsychological assessments—and structural imaging (Table 3).

**TABLE 3:** Investigations performed prior to referral for the false‐positive patients (*n* = 66).

Test	Investigations completed prior to referral (*n*=)	Investigations that caused misdiagnosis (*n*=)
Structural imaging		
CT	52	4
MRI	57	13
Nuclear imaging		
HMPAO‐SPECT	25	20
FDG‐PET	27	12
Cognitive testing		
Bedside tests	26	9
Neuropsychology	26	20

Abbreviations: CT, computed tomography; FDG‐PET, fluorodeoxyglucose positron emission tomography; HMPAO‐SPECT, hexamethylpropyleneamine oxime single‐photon emission computed tomography; MRI, magnetic resonance imaging.

**FIGURE 2:**
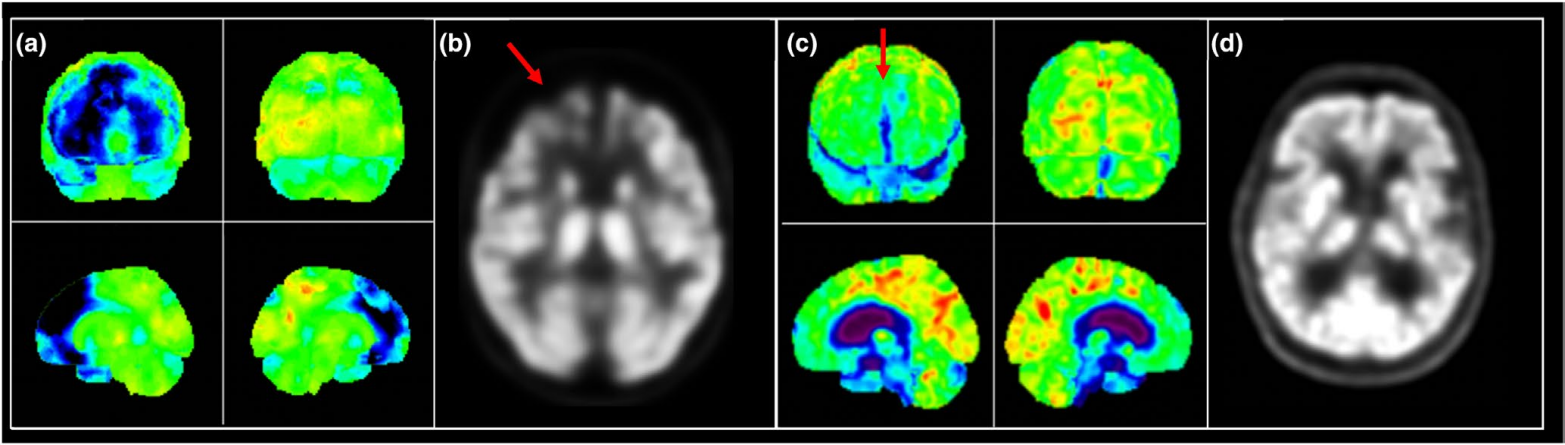
FDG‐PET scans of a true‐ and false‐positive patient both reported to have frontal lobe hypometabolism suggestive of bvFTD. (a) Stereotactic surface projection of a true‐positive bvFTD patient showing obvious frontal lobe hypometabolism and their grey‐scale image also showing obvious frontal lobe hypometabolism (b, arrow). (c) Stereotactic surface projection of a false‐positive patient who did not meet criteria for a clinical disorder. A registration artefact within the interhemispheric fissure was misinterpreted as hypometabolism (arrow) while their grey‐scale image showed normal glucose metabolism (d).

The main factors leading to misinterpretation of nuclear imaging included: (a) subjective interpretation of frontotemporal hypoperfusion/hypometabolism without quantitative statistical maps (e.g. stereotactic surface projection software), (b) registration errors causing artefacts in statistical maps (Figure 2). Notably, *n* = 20/30 patients who were ultimately diagnosed with a psychiatric disorder were initially suspected of having bvFTD based on nuclear imaging.

Regarding cognitive test performance, executive dysfunction or working‐memory deficits with relative sparing of other cognitive domains was the primary justification for suspecting bvFTD in false‐positive patients. Full neuropsychology assessments were available for *n* = 12 true‐positive and *n* = 16 false‐positive patients. Impaired performance on executive function tests was common for both true‐positive and false‐positive patients (83.3% vs. 87.5%, *χ* ^2^ 0.097, *p* = 0.755), whereas relative preservation of performance on memory and visuospatial tests was much less likely for true‐positive compared with false‐positive patients (8.3% vs. 62.5%, *χ* ^2^ 8.435, *p* = 0.004). Accordingly, the Rascovsky cognitive profile of bvFTD—executive dysfunction with relatively intact memory and visuospatial skills—was much less likely for true‐positive compared with false‐positive patients (8.3% vs. 50.0%, *χ* ^2^ 5.458, *p* = 0.019).

Structural neuroimaging reporting focal frontotemporal atrophy at a single time‐point contributed to a false‐positive bvFTD diagnosis in *n* = 6/30 patients in the psychiatric cohort. This was also the case in *n* = 2/8 patients that did not meet criteria for a clinical disorder. In these patients, ‘frontotemporal atrophy’ was reported as an incidental finding to the reason for the scan being requested, and yet was the catalyst for a diagnosis of bvFTD despite these patients lacking any clinical features of bvFTD. A misreport of focal frontotemporal atrophy led to a false‐positive bvFTD diagnosis in *n* = 3 patients with clinical, biomarker‐confirmed Alzheimer's disease. Arguably in each instance, atrophy was more diffuse than the reports implied compared with true‐positive patients.

Structural MRI also correctly identified focal frontal lobe atrophy in *n* = 3 patients who had clear clinical evidence of an organic frontal lobe syndrome—though not due to an FTLD pathology. These three were as follows: a genetically confirmed CSF1R‐leukoencephalopathy patient (also had confluent frontal white matter hyperintensities); a genetically confirmed Huntington's disease patient (with only subtle chorea); and a patient with autoimmune encephalitis (with CSF confirmation of NMDAr antibodies). All three patients had Rosovsky's behavioural profile for bvFTD, with focal frontal lobe atrophy confirmed by serial neuroimaging—though met exclusion criteria due to the confirmation of an alternative neurological disorder that better explained their symptoms.

Diagnostic dilemmas accounted for *n* = 5 false‐positive diagnoses with each displaying non‐specific behavioural symptoms (e.g. ‘aggression’) and neuroimaging reported as normal. This cohort were heterogenous; with longitudinal follow‐up, detailed psychiatric interviewing and further investigations often leading to an alternative diagnosis to explain their clinical symptoms. Common psychiatric and psychosocial explanations for these individuals included psychosocial stressors precipitating presentation to medical services; alcohol and illicit substance use; and previously unidentified features of developmental disorders (e.g. autism and intellectual disabilities). Often, the psychosocial stressor or substance use was minimized by the patient, or their informant, at initial presentation—thus leading to an impression of bvFTD.

Finally, there were *n* = 3 patients in whom a family history of a disease in the FTLD spectrum led to a false‐positive diagnosis of bvFTD. None met Rascovsky criteria for even possible bvFTD; instead, they presented with non‐specific cognitive and behavioural symptoms and were led to a diagnosis of bvFTD purely based on family history of FTD‐MND, GRN‐positive bvFTD or PSP.

#### Mater clinic assessment: Comparison to final diagnoses

**FIGURE 3:**
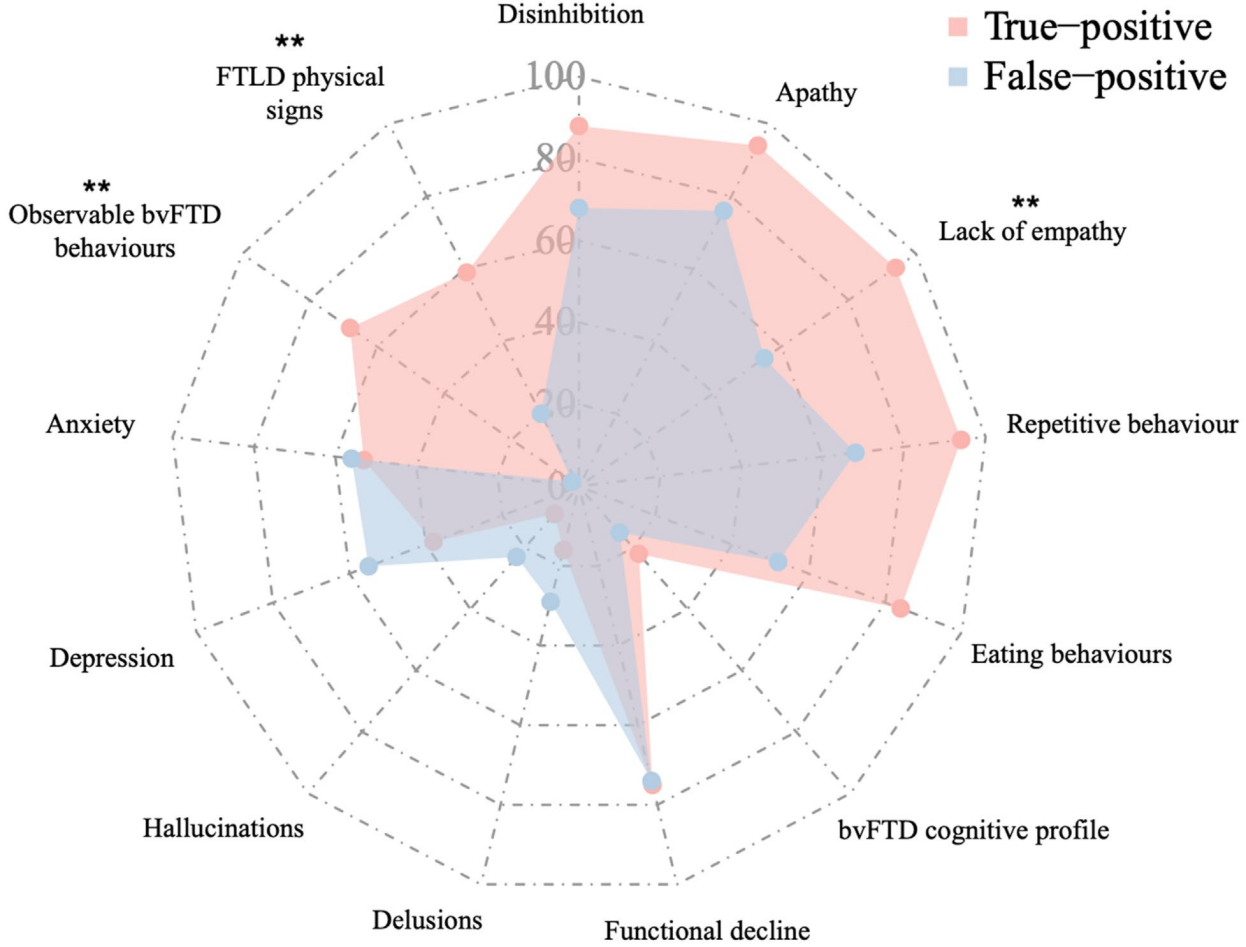
Radar graph of clinical data from the baseline Mater Clinic assessments. bvFTD: behavioural variant frontotemporal dementia. Statistically significant differences between cohorts **p < 0.01.

The prevalence of clinical features in true‐ versus false‐positive patients are summarized in Figure 3 with further analyses stratifying false positives into subgroups in Table 4. Each of the behavioural features of bvFTD—apathy, empathy loss, repetitive behaviours, disinhibition and eating behaviours—occurred with over 80% prevalence in the true‐positive group; all of these behaviours were more common in true positives compared with false positives, but this only reached statistical significance for lack of empathy. In contrast, the cognitive profile of bvFTD, based on sub‐tests from the ACE‐III, was uncommon and present at a similar rate in both true‐positive and false‐positive patients. Functional decline was common but present at a similar rate in both true‐positive and false‐positive patients. All psychiatric symptoms were less common in true‐positive compared with false‐positive patients, but this was not statistically significant. The most striking differences were highly significant increased prevalence of observable behavioural features of bvFTD and physical neurological signs in true‐positive patients. These behavioural features included disinhibition (e.g. impulsive behaviour and verbal disinhibition), repetitive behaviours (e.g. echolalia, echopraxia, palilalia, stereotypic movements, use of catchphrases and verbal perseveration), apathy (e.g. emotional blunting, dynamic aphasia and inertia), utilization behaviour and a fatuous affect. The physical neurological signs included those of PSP, CBS or MND.

**TABLE 4:** Mater clinic assessments: Comparison in prevalence of clinical features between cohorts.

Clinical feature	True‐positive bvFTD(%)	Psychiatric disorders(%)	Neurological disorders(%)	No disorder	Omnibus significance (*χ* ^2^)
Disinhibition	87.5	72.4	60.7	80.0	ns
Apathy	93.8	69.0	60.7	80.0	ns
Empathy loss	93.8	65	35.7^a^	100.0	*χ* ^2^ 25.635, *p* < 0.001*
Repetitive behaviours	93.8	69.0	60.7	100.0	*χ* ^2^ 11.662, *p* = 0.009
Eating behaviours	84.4	48.3	57.1	66.7	*χ* ^2^ 10.487, *p* = 0.015
BvFTD cognitive profile	21.9	20.0	10.7	14.3	ns
Functional decline	75.0	72.4	75.0	80.0	ns
Delusions	15.6	24.1	35.7	20.0	ns
Hallucinations	9.4	27.6	21.4	0.0	ns
Depression	37.5	58.6	57.2	20.0	ns
Anxiety	53.1	51.7	57.1	80.0	ns
Observable bvFTD behaviour	67.6	0.0^a^	7.1^a^	0.0^a^	*χ* ^2^ 50.409, *p* < 0.001*
Physical neurological signs	52.9	13.3^a^	28.6	0.0^b^	*χ* ^2^ 15.945, *p* < 0.001*

* Significant after Bonferroni correction for multiple comparisons. Superscripts (^a^, ^b^) indicate statistically significant differences between bvFTD and the other cohorts in the post hoc analysis (*χ* ^2^)

Abbreviations: bvFTD, behavioural variant frontotemporal dementia; FTLD, frontotemporal lobar degeneration; ns, no significance.

^a^ *p* < 0.001

^b^ *p* < 0.01

Stratifying the false‐positive group into psychiatric, neurological and no clinical disorder groups, showed that false‐positive patients with observable bvFTD behaviours were only found in the neurological subgroup (Table 4). These were two patients with organic frontal lobe syndromes—one with a prefrontal stroke and the other with autoimmune encephalitis. These behaviours were never observed in the psychiatric and no clinical disorder groups. Physical neurological signs were observed in both the psychiatric and neurological subgroups. In the psychiatric group, these were all considered iatrogenic (either parkinsonism in patients taking antipsychotics or myoclonus in patients taking multiple or high dose antidepressants). A miscellany of physical signs was observed in the neurological group compatible with their underlying, specific diagnoses. Behavioural features of bvFTD and functional decline were each reported with at least 80% prevalence by informants in the group with no clinical disorder.

## DISCUSSION

The findings of this study emphasize the significant diagnostic challenges associated with bvFTD. Despite the referrals only coming from specialist physicians, the rate of false‐positive diagnoses was substantial, with 66% of referred patients ultimately not meeting the criteria for bvFTD. This high rate of misdiagnosis highlights the complexities in differentiating bvFTD from psychiatric and other neurological conditions, particularly when relying on diagnostic tools, such as nuclear imaging and cognitive testing.

Misinterpretation of nuclear imaging, specifically HMPAO‐SPECT and FDG‐PET scans, was identified as a major source of diagnostic error. An erroneous bvFTD diagnosis frequently related to subjective interpretation of frontotemporal hypoperfusion or hypometabolism, often without quantitative confirmation. This finding is consistent with previous research, which emphasized that while imaging can be a valuable tool for diagnosing bvFTD, it requires careful and accurate interpretation—qualitative visual assessments by radiologists have shown low specificity in diagnosing bvFTD [13, 14] and have been outperformed by quantitative assessment methods [15]. However, automated approaches that generate statistical maps were also the cause of false‐positive diagnoses in some instances. In particular, artefacts on the mesial frontal surfaces, likely caused by misregistration to templates, were mistakenly reported as hypometabolism. This finding highlights that while automated approaches have advantages over qualitative visual interpretation of raw images, these methods also require technical knowledge of limitations and pitfalls [16].

Cognitive testing, especially when indicating executive dysfunction with sparing of memory and visuospatial skills, was another significant factor contributing to false‐positive diagnoses. This profile, although proposed as characteristic of bvFTD in the Rascovsky criteria, can overlap with psychiatric disorders, complicating the diagnostic process. Previous systematic reviews have demonstrated that the most common psychiatric disorders considered in the differential diagnosis of bvFTD are often associated with isolated executive dysfunction and/or social cognition deficits [17, 18, 19, 20]. The present study demonstrated that this cognitive profile was statistically more likely to occur in the psychiatric cohort than in the true‐positive cohort. It was also interesting that in the true‐positive bvFTD patients, a profile of executive dysfunction with relative sparing of memory and visuospatial skills was rare. Notably, none of the neuropsychology reports reviewed in this study included social cognition testing. Tests of social cognition have been shown to outperform traditional tests of executive function in identifying bvFTD [21], and it has been proposed by an expert panel that such tests should be incorporated into clinical assessment [14]. Whether inclusion of such tests would have improved diagnostic accuracy in the present series; however, one can only speculate.

Patients with a prior psychiatric history or those prescribed psychiatric medications were statistically more likely to be misdiagnosed with bvFTD. This finding underscores the overlapping symptomatology between bvFTD and primary psychiatric disorders, which can lead to diagnostic confusion. This is highlighted in previous studies, which have shown the poor specificity of possible bvFTD diagnostic criteria [22]. Physicians should exercise caution in diagnosing bvFTD in patients with chronic psychiatric disorders, and moreover, probe carefully for a past psychiatric history—such history was sometimes downplayed by patients and informants in the present series. Overlapping symptom profiles were also seen between bvFTD and false positives due to an alternate neurological diagnosis. This was unsurprising given that several of these patients had organic frontal lobe pathology from other aetiologies.

While the characteristic behavioural symptoms of bvFTD were highly prevalent in true‐positive patients, this behavioural profile was particularly evident in patients who were ultimately concluded to have no clinical disorder at all. Given that bvFTD had been proposed by the referring physicians in the false‐positive patients, it seems conceivable that confirmation bias on the part of informants might have contributed to these symptom endorsements. This does not necessarily imply that informants were wilfully trying to mislead. For example, once a diagnosis of bvFTD had been suggested, behaviours, such as making a rude remark, acting selfishly, eating chocolate—that is behaviours that can be observed in the general population—may take on new significance for an informant. It seems plausible that this phenomenon would be particularly exacerbated when the diagnosis was supported by the report of a brain scan or neuropsychological assessment—investigations that may be perceived as objective and irrefutable by patients and informants.

Empirically observed behavioural features of bvFTD in the clinic were significantly less common in false‐positive patients during initial assessments. The only false‐positive patients with observed behavioural features of bvFTD were patients with organic frontal lobe syndromes from other aetiologies. Although the behavioural profile of bvFTD is well documented, little attention has been given in research studies to who has provided this information. This discrepancy suggests that physicians should be wary of relying solely on subjective reports from informants. In contrast, greater emphasis should be placed on objective behaviours observed during clinical examination. However, notably, while observed behaviours were highly specific for bvFTD, they were not observed in one‐third of true‐positive cases at initial assessment. Additionally, the emergence of neurological features of FTLD with longitudinal follow‐up was almost exclusively found in the true‐positive cohort. The only patients within the false‐positive cohort that had neurological features of FTLD documented were within in the neurological subgroup.

Despite the strengths of this study, including its focus on a well‐defined, consecutive cohort of patients with suspected bvFTD, limitations must be acknowledged. The lack of histopathological confirmation for all patients is a significant constraint, as this remains the gold standard for definitive diagnosis of neurodegenerative diseases [23]. However, the emphasis on longitudinal follow‐up with documentation of functional decline and progressive focal frontotemporal volume loss mitigates the risk of incorrect diagnoses in the current series [22]. The another key limitation was that the information on which referring physicians based their diagnoses was not collected in a systematic manner. Thus, the findings in this study cannot be used to infer metrics, such as predictive values, sensitivity or specificity of different investigations; rather, the data offer a pragmatic snapshot of common themes in misdiagnosis.

## CONCLUSION

False‐positive diagnosis of bvFTD remains exceedingly prevalent. However, on an optimistic note, the results suggested that the number of misdiagnoses could be considerably reduced by improving awareness of the dangers of over‐interpretation of neuroimaging and neuropsychological assessments. Physicians must remain especially vigilant to the presence of past psychiatric illness and place particular weight on objective behavioural observations and neurological examination during assessments.

## AUTHOR CONTRIBUTIONS

**Joshua Flavell:** Conceptualization; data curation; formal analysis; investigation; methodology; project administration; resources; software; validation; visualization; writing – original draft; writing – review and editing. **Emily G. M. Ahern:** Conceptualization; visualization; writing – review and editing. **Benignus Logan:** Visualization; writing – review and editing. **Thomas B. Shaw:** Formal analysis; validation; visualization; writing – review and editing. **Robert J. Adam:** Visualization; writing – review and editing. **Caitlin A. T. McElligott:** Data curation; resources; writing – review and editing. **Peter J. Nestor:** Conceptualization; data curation; formal analysis; funding acquisition; investigation; methodology; supervision; validation; visualization; writing – original draft.

## FUNDING INFORMATION

We are grateful for the financial support from the Mater Foundation and the Vonnie Healy Research Grant.

## CONFLICT OF INTEREST STATEMENT

None.

## DATA SHARING AND DATA ACCESSIBILITY

There are ethical restrictions in place for sharing de‐identified data. The ethics approved for this project allows for the sharing of de‐identified data between institutions included in the study (Mater Misericordiae). The data include sensitive clinical information relating to the patients' signs, symptoms, medical history, investigations and cognitive assessments. The data reported in the article can be shared with other research groups with appropriate ethical approval by contacting: research.ethics@mater.uq.edu.au.

## ETHICS STATEMENT

This study was approved by the Mater Misericordiae Ltd. Human Research Ethics Committee (EC00332) as a retrospective chart review, with a waiver of informed consent granted in accordance with local ethical guidelines.

## Data Availability

The data that support the findings of this study are available on request from the corresponding author. The data are not publicly available due to privacy or ethical restrictions.
